# Factors associated with weight Z-score in very low birth weight and extremely low birth weight preterm infants during hospitalization

**DOI:** 10.1016/j.jped.2025.101443

**Published:** 2025-09-25

**Authors:** Eduarda Couto Plácido Nunes, Eduarda Silva, Eduarda Dallmann Lopes Pereira, Karla Pereira Machado, Sandra Costa Valle

**Affiliations:** a Universidade Federal de Pelotas (UFPel), Pelotas, RS, Brazil; b Universidade Federal de Pelotas (UFPel), Faculdade de Nutrição, Pelotas, RS, Brazil

**Keywords:** Premature newborn, Very low birth weight infant, Extremely low birth weight infant, Weight gain trajectory, Energy, Protein

## Abstract

**Objective:**

To investigate the behavior of weight-for-age z-score (WAZ) and associated factors in preterm newborns (PTNB) with very low birth weight (VLBW) and extremely low birth weight (ELBW), comparing them to low birth weight (LBW) during four weeks (wk.) of hospitalization in a neonatal intensive care unit (NICU).

**Methods:**

Longitudinal study conducted using data from PTNB records in the southern Brazil NICU, between January 2017 and December 2020. Non-twin PTNB with gestational age of ≥ 24 and < 37 wk. and a birth weight (BW) ≥ 500 g were included. The outcome was WAZ, and the exposure was the PTNB’s BW, categorized as VLBW/ELBW (< 1500 g) and LBW (≥ 1500 g up to 2500 g). Energy (kcal/kg/day) and protein (g/kg/day) intakes were also evaluated. Two-way ANOVA and multiple linear regression were used to assess the association between demographic, clinical, and nutritional factors and the WAZ.

**Results:**

The majority of PTNB were male (60 %) and had a birth weight ≥1500 g (65 %). A significant interaction was between the BW category and the length of hospitalization on WAZ (*F* = 4.0; *p* = 0.003). In the VLBW and ELBW, the WAZ was significantly lower in the first wk. compared to the LBW [−1.05 (−1.34;−0.75) vs −0.34 (−0.49;−0.18)]. Factors such as male sex, sepsis, initiation of enteral nutrition (EN), and protein intake were associated with WAZ behavior.

**Conclusion:**

The downward trend of the WAZ curve was associated with the interaction between birth weight and length of hospitalization, especially in PTNB with VLBW and ELBW. The study concluded that male sex and sepsis contributed to the observed decline.

## Introduction

Prematurity is a global public health issue, accounting for 9.9 % all births in 2020 [1]. In Brazil, prematurity is the leading cause of mortality in children under five years old, with a similar rate [1,2]. Preterm newborns (PTNB) with very low birth weight (VLBW) and extremely low birth weight (ELBW) constitute 15 % of this rate and require increased attention regarding their growth and associated factors, particularly in early postnatal life [2,3].

Technological advancements in extrauterine life support and care practices have reduced morbidity and mortality in PTNB, especially in the VLBW and ELBW groups, which are frequently linked to lower gestational age (GA) [3]. Despite this progress, these groups show a high prevalence (24 %–53 %) of negative neurological outcomes, such as cognitive, motor, auditory, and visual deficits, cerebral palsy, and communication difficulties [3, 4, 5]. They also have a higher prevalence of growth deficits at hospital discharge and during early childhood, alongside an increased risk of chronic diseases in the long term [6,7].

In the neonatal intensive care unit (NICU), the growth pattern for preterm infants with VLBW and ELBW is based on fetal growth [8]. However, due to high nutritional requirements, insufficient nutrient provision/utilization, and the pathophysiology of morbidities, the accumulation of growth deficit occurs during hospitalization. Thus, understanding and improving the dynamics of in-hospital growth of these PTNB is crucial, given its relationship with neurodevelopment and the burden of diseases attributed to prematurity [8,9].

Monitoring the nutritional status of PTNB allows for the early identification of nutritional deficits and timely adjustments to nutritional support, with the aim of mitigating negative short- and long-term outcomes [10, 11, 12]. However, PTNB often experience weight loss, corresponding to a reduction of approximately 0.8 z-score compared to birth weight (BW), which establishes a new growth curve parallel to the intrauterine one [10,13]. The analysis of this new growth curve’s behavior must consider additional factors to determine if these PTNB are recovering from deficits accumulated during their hospital stay [10,13].

The prognosis of PTNB depends on factors such as gestational age (GA), morbidities, and neonatal and nutritional care practices [2,8]. Additionally, the adaptive response to extrauterine life may be intrinsically different. For example, female PTNB have shown a better response to nutritional support and a lower rate of morbidity and mortality compared to males [14].

A retrospective study evaluating the impact of a higher and more rapid energy and protein supply on the growth velocity of extremely preterm infants hospitalized in the NICU concluded that a positive energy and protein balance was essential for adequate postnatal growth and for the prevention of extrauterine growth restriction (EUGR) [15].

The study’s aim was to investigate the behavior of the weight-for-age z-score (WAZ) and associated factors in PTNB with VLBW and ELBW, comparing them to PTNB with LBW, over four weeks of hospitalization in a NICU.

## Methods

This analytical, retrospective, longitudinal study utilized medical record data from PTNB admitted within their first 48 h of life to the NICU of a university hospital in southern Brazil, between January 2017 and December 2020. The NICU admits an average of 18 patients monthly, primarily from the southern region.

For this study, PTNB of both sexes, non-twins, with a gestational age ≥ 24 and < 37 weeks, and a birth weight (BW) ≥ 500 g, receiving nutrients via parenteral/enteral routes, were included. Newborns with neonatologist diagnoses capable of impacting growth, anthropometry, and/or nutrition, such as microcephaly and hydrocephaly, chromosomal abnormalities, fetal hydrops, heart disease, gastroschisis, and congenital malformations, were excluded. Patients with missing anthropometric data records during the study period were also excluded.

**Figure 1:**
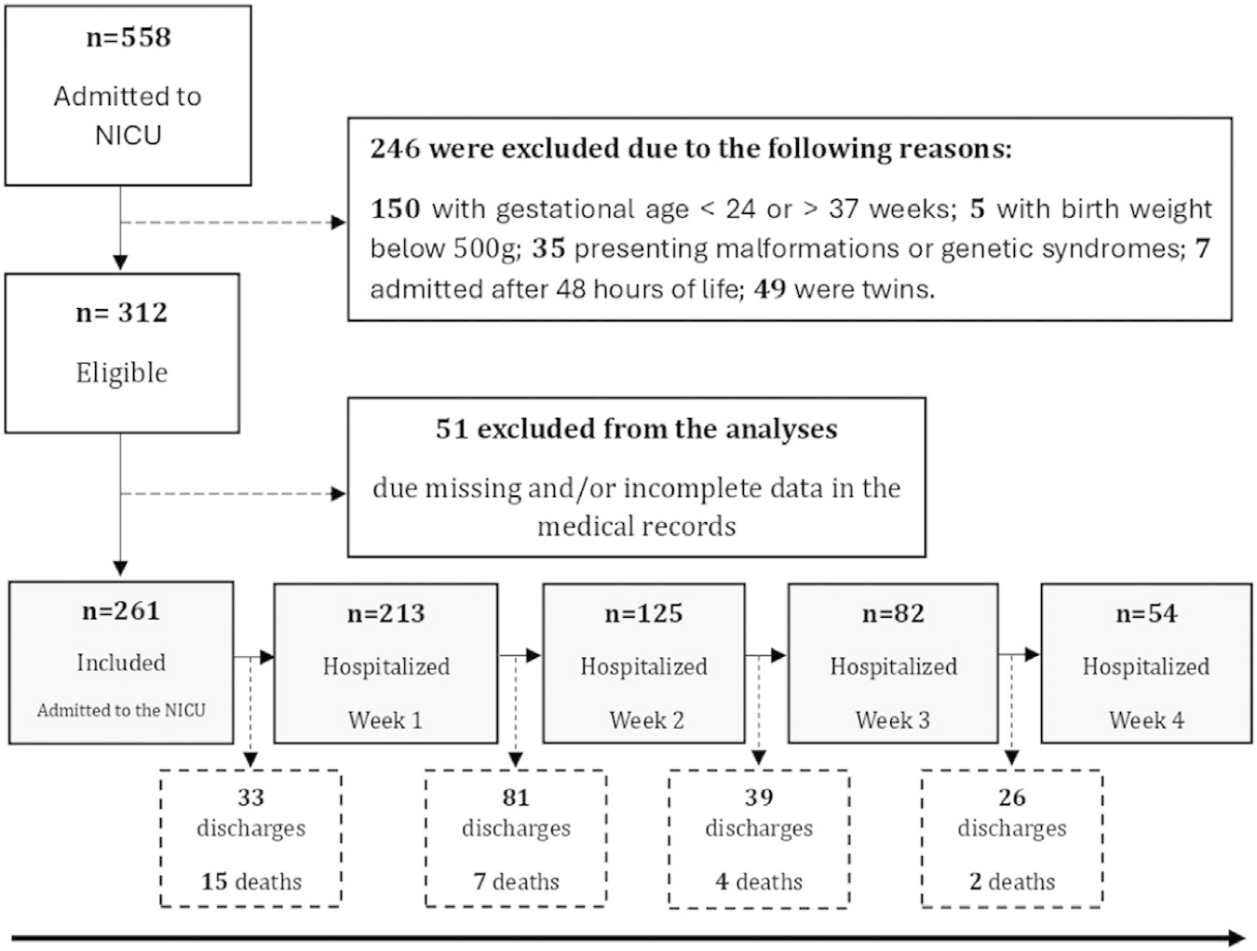
Study Flow Diagram.

A total of 558 PTNB records were obtained; 297 were excluded (29 deaths), leaving 261 for this study. Data from eligible preterm infants were categorized into five phases based on days of hospitalization: Admission (birth up to 48 h), Week 1 (5–7 days), Week 2 (12–14 days), Week 3 (19–21 days), and Week 4 (26–28 days). Of the included PTNB, 213, 125, 82, and 54 remained hospitalized in the NICU during weeks 1, 2, 3, and 4, respectively (Figure 1). This grouping strategy was established to capture critical periods impacting growth and to ensure an adequate sample size for the analyses.

The GA was estimated based on the following priority order: i) maternal report of the date of the last menstrual period, if it did not differ by no more than two weeks from the GA provided by fetal ultrasonography performed up to the 20th week of gestation; ii) fetal ultrasonography performed up to 20 weeks of gestation, in cases where maternal GA was deemed unrealiable and the difference between ultrasonographic and the New Ballard scores was less than two weeks; or iii) postnatal GA calculated by the New Ballard Score method, when there was a difference greater than two weeks from maternal and ultrasonographic GA.

The sociodemographic, clinical, anthropometric, and nutritional data were recorded by NICU nutritionists during routine follow-up. Anthropometry was performed by technical nurses who received prior and systematic training from the nutrition team, according to the recommendations described by the World Health Organization (WHO) in the Anthropometry Handbook [16].

The primary outcome was the WAZ, which was calculated using the Intergrowth-21st online calculators for Postnatal Growth of Preterm Infants based on postmenstrual age and sex. To ensure data reliability, all calculations and z-score entries were independently verified by two researchers. In cases of discrepancies, original physical forms were consulted, and the calculations were reviewed.

The main exposure was BW according to WHO criteria [17]. For this purpose, PTNB were categorized into two groups: 1) Very low birth weight and Extremely low birth weight (VLBW and ELBW): BW < 1500 g and 2) Low birth weight (LBW): BW ≥ 1500 g up to 2500 g. The VLBW and ELBW groups were combined to ensure an adequate sample size for statistical analyses.

Energy (kcal/kg/day) and protein (g/kg/day) intakes administered during the follow-up period were evaluated. The daily content of energy nutrients (g/mL) from parenteral nutrition (PN) and/or enteral nutrition (EN) solutions was obtained according to the NICU prescription. The volume considered for the solutions was the amount effectively administered in 24 h. The daily energy and protein intake was calculated following the nutritional information on the labels of Parenteral Nutrition (PN) solutions or products used via enteral nutrition (EN) (preterm infant formulas (PIF), human milk fortifiers (HMF)). In the case of human milk (HM) administration, the nutritional composition of preterm HM was considered for the calculation, according to the week postpartum, based on information from the Ministry of Health [18].

Weight gain velocity (g/kg/day) was calculated up to the fourth week of hospitalization using the equation proposed by Fenton et al. [19]: [(current weight – previous weight) /([(previous weight + current weight)/2]/1000)/number of days], with weight in grams from the lowest weight (weight nadir) during the neonatal period.

Weight in grams was measured using a Filizola Baby® electronic scale (with an approximation of 5 g) after taring, discounting any equipment attached to the newborn. Length was measured in centimeters using a SECA 210 portable anthropometer (with 5 mm graduations), with the newborn in the dorsal decubitus position on a horizontal Frankfurt plane, with the cephalic end fixed and the podalic end mobile, with the assistance of another person to restrain the neonate. Head circumference was measured in centimeters with a non-stretchable measuring tape (with an approximation of 0.1 cm), considering the largest occipitofrontal diameter. The absolute values of Z-scores for length and head circumference, in relation to age and sex, were obtained using the Intergrowth-21st calculators.

The other covariates were GA collected in weeks and days, sex (female/male), length of hospitalization (days), APGAR score (1 to 10 points), sepsis during follow-up (yes/no), respiratory distress syndrome (yes/no), jaundice (yes/no), other diseases (yes/no), time to initiation of EN (hours), weight nadir (g), time to reach nadir (days), WAZ at nadir, percentage of weight loss at nadir obtained by the equation: {[(current weight - previous weight) / previous weight] x 100}), receiving HM (yes/no), intercurrent events (yes/no), and Z-scores for length and head circumference.

Statistical analyses were performed using JAMOVI software, version 2.5. Results are presented as relative and absolute frequencies, means, and 95 % confidence intervals (95 %CI, min;max). The variable’s distribution was checked by the Shapiro-Wilk test. When symmetrical, the categories were performed by Student's *t*-test, when asymmetrical by Mann-Whitney U test. Two-way ANOVA, followed by Bonferroni post-hoc test, was applied to compare the effect of BW category according to the length of hospitalization and the interaction between these two factors on the trajectory of WAZ, energy and protein intake, and weight gain.

Multiple linear regression analysis was used to investigate the association between demographic, clinical, and nutritional factors and the behavior of WAZ in PTNB with VLBW and ELBW compared to LBW. Independent variables were individually assessed using bivariate linear regression, and those with a p-value > 0.2 were included in the multiple linear regression model. Initially, the global significance test of the model was performed. From a significant F-value, the verification of autocorrelation, multicollinearity, and normality was carried out. If these assumptions were met, the standardized coefficients and adjusted R² were evaluated. The explanatory variables maintained in the multiple regression model were: sex, sepsis, time to initiation of EN, and protein intake. A p-value < 0,05 was adopted.

This study was approved by the Research Ethics Committee of the Faculty of Nursing, CAAE 80,543,424.4.0000.5316, under opinion number:6.961.286, through Plataforma Brasil.

## Results

Of the 261 PTNB in this study, 60 % were male, and 65 % were born with a weight ≥ 1500 g. Regarding morbidities, 80 % presented with respiratory distress syndrome, while 41 % and 27 % had sepsis and jaundice, respectively (data not presented in tables).

Table 1 presents the clinical and nutritional characteristics of the sample, according to the BW category. The group of preterm infants born with VLBW and ELBW, when compared to the LBW group, had lower GA [29.5 (29;30) vs 34.0 (33.6;34.3) weeks and days], longer length of hospitalization [45.3 (38.1;52.4) vs 13.4 (11.1;15.6) days], lower WAZ at birth [−0.32 (−0.61;−0.02) vs 0.56 (0.41;0.71), WAZ] and at weight nadir [−1.19 (−1.53;−0.84) vs −0.30 (−0.49;−0.12), WAZ], and shorter time to initiate PN [12.9 (10.2;15.6) vs 24 (10.7;37.3) hours]. At birth, the VLBW and ELBW group had lower weight [1118 (1066;1171) vs 2276 (2199;2352) g], head circumference (HC) [26.1 (25.6;26.6) vs 31.5 (31.2;31.8) cm], and length [36 (35.4;36.7) vs 44 (43.5;44.4) cm].

**Table 1:** Clinical and Nutritional Characteristics According to Birth Weight Category in Preterm Newborns Admitted to a Neonatal Intensive Care Unit. University Hospital– Federal University of Pelotas (UFPEL). *N* = 261, 2017–2020.

**Variable**	**All** **(*n*=261)**	**LBW** **(*n*=170)**	**VLBW and ELBW** **(*n*=91)**	**p-value**
Gestational Age (weeks)	32.5 (32.1–32.8)	34.0 (33.6–34.3)	29.5 (29.0–30.0)	<0.001*
Length of Hospital Stay (days)	23.2 (19.9–26.5)	13.4 (11.1–15.6)	45.3 (38.1–52.4)	<0.001*
Weight (g)	1872.0 (1786.0–1958.0)	2276.0 (2199.0–2352.0)	1118.0 (1066.0–1171.0)	<0.001*
Head Circumference (cm)	29.6 (29.2–30.0)	31.5 (31.2–31.8)	26.1 (25.6–26.6)	<0.001**
Length (cm)	41.2 (40.6–41.8)	44.0 (43.5–44.4)	36.08 (35.4–36.7)	<0.001**
Weight Loss at Nadir ( %)	6.3 (5.7–6.9)	6.0 (5.3–6.7)	6.8 (5.6–7.9)	0.256
Time to Weight Nadir (days)	5.0 (4.7–5.3)	4.9 (4.6–5.3)	5.2 (4.8–5.7)	0.254
Weight at Nadir (g)	1770.0 (1669.0–1872.0)	2120.0 (2023.0–2217.0)	1071.0 (1019.0–1115.0)	<0.001*
Z-score at Birth	0.25 (0.10–0.40)	0.56 (0.41–0.71)	–0.32 (–0.61 to –0.02)	<0.001*
Z-score at Nadir	–0.62 (–0.80 to –0.44)	–0.30 (–0.49 to –0.12)	–1.19 (–1.53 to –0.84)	<0.001**
Time to PN Start (hours)	13.8 (11.1–16.5)	24.0 (10.7–37.3)	12.9 (10.2–15.6)	0.044*
Time to EN Start (hours)	16.7 (13.4–20.0)	15.4 (11.7–19.1)	19.1 (12.7–25.4)	0.178
Initial EN (kcal/kg/day)	10.4 (8.1–12.7)	9.6 (7.7–11.6)	12.4 (5.8–19.0)	0.314

LBW, Low Birth Weight (≥ 1500 g and < 2500 g); VLBW, Very Low Birth Weight (< 1500 g); ELBW, Extremely Low Birth Weight (< 1000 g); PN, Parenteral Nutrition; EN, Enteral Nutrition; CI, Confidence Interval.

⁎ *p* < 0.05.

⁎⁎ *p* < 0.001.

**Figure 2:**
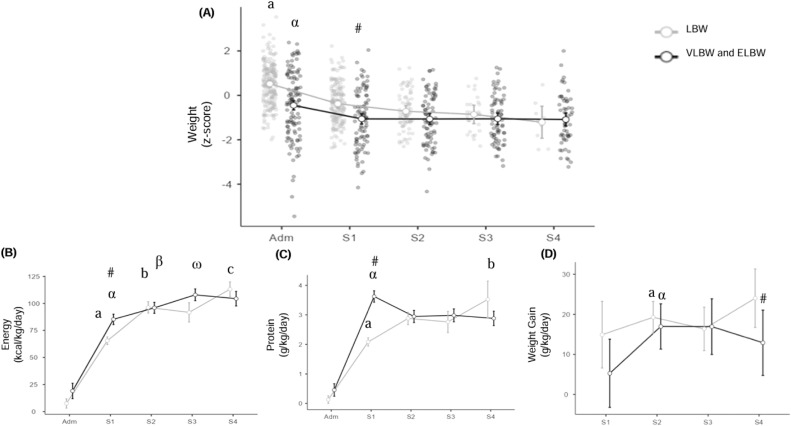
(A) Weight Z-score trend, (B) Energy intake (kcal/kg/day), (C) Protein intake (g/kg/day), (D) Weight gain (g/kg/day), according to birth weight category, of preterm newborns admitted to a Neonatal Intensive Care Unit. University Hospital-UFPEL. *N* = 261, 2017–2019. LBW, Low birth weight; VLBW and ELBW, Very low birth weight and Extremely low birth weight. Adm: admission; W1, W2, W3, and W4, weeks of hospitalization 1, 2, 3, and 4, respectively. Two-way ANOVA. Roman letters indicate *p* < 0.05 between weeks of hospitalization in the LBW group; Greek letters indicate *p* < 0.05 between weeks of hospitalization in the VLBW and ELBW group; # indicates *p* < 0.05 between birth weight categories, Bonferroni test.

Regarding WAZ, a significant interaction was observed between BW category and length of hospitalization on the curve's behavior (*F* = 4.0; *p* = 0.003). There was also a significant effect of BW category (*F* = 14.9; *p* < 0.001) and length of hospitalization (*F* = 36.6; *p* < 0.001) on the reduction of WAZ. In the VLBW and ELBW group, the WAZ was significantly lower in the first week of hospitalization compared to the LBW group [−1.05 (−1.34;−0.75) vs −0.34 (−0.49;−0.18), WAZ] (Figure 2A and Supplementary Table I).

Concerning energy intake, a significant interaction (*F* = 8.6; *p* < 0.001) and significant association were observed between BW category (*F* = 15.9; *p* < 0.001) and length of hospitalization (*F* = 316.7; *p* < 0.001) on the increase in energy intake. In the VLBW and ELBW group, energy intake was significantly higher in the first week of hospitalization compared to the LBW group [85.3 (80.8;89.8) vs 65.5 (61.3;69.7) kcal/kg/day] (Figure 2B).

Regarding protein intake, a significant interaction (*F* = 23.3; *p* < 0.001) and significant association were found between birth weight category (*F* = 10.7; *p* < 0.001) and length of hospitalization (*F* = 295.7; *p* < 0.001) on the increase in protein intake. The comparison between groups showed a significant increase in protein intake in the first week of hospitalization in the VLBW and ELBW group compared to the LBW group [3.6 (3.4;3.8) vs 2.1 (1.8;2.26) g/kg/day] (Figure 2C).

Weight gain velocity was significantly lower in VLBW and ELBW PTNB compared to those with LBW (*F* = 5.1; *p* = 0.025), corresponding to an average of 14.2 g/kg/day (12.6;15.5) and 18.7 g/kg/day (15.3;22.1), respectively. Considering the weeks of hospitalization, in the VLBW and ELBW group, weight gain velocity was significantly lower only in the fourth week of hospitalization compared to the LBW group [12.9 (9.6;16.2) vs 24.0 (10.3;37.8) g/kg/day] (Figure 2D).

In the unadjusted linear regression analysis, WAZ was lower in VLBW and ELBW PTNB compared to LBW PTNB [B −0.70 (−0.89;−0.35), adjusted R² 8.7 %, *F* = 21.5; *p* < 0.001]. In the analysis adjusted for male sex, sepsis during hospitalization, time to initiation of EN, and protein intake (g/kg/day), the observed difference remained significant [B −0.40 (−0.70;−0.01), adjusted R² 19 %, *F* = 9.19; *p* < 0.001]. The variability of WAZ explained by the set of variables in the model increased 2.4 times compared to that observed in the crude analysis for VLBW and ELBW preterm infants (Table 2 and Supplementary Fig. 1).

**Table 2:** Linear regression of Weight Z-score, according to birth weight category, of preterm newborns admitted to a Neonatal Intensive Care Unit. University Hospital-UFPEL, 2017–2019.

**Birth weight category**	**Weight z-score**									
	**Unadjusted analysis**					**Adjusted analysis^a^**				
	B	β	IC 95 %	R^2^	*p*	B	β	IC 95 %	R^2^	*p*
Low Birth Weight	Ref.					Ref				
Very Low and Extremely Low Birth Weight	−0.70	−0.62	−0.89;0.35	8.7	<0.001	−0.40	−0.35	−0.7;−0.01	19.0	<0.001

B, intercept; β, standardized coefficient; 95 % CI, 95 % confidence interval; R², adjusted R² ( %). *N* = 177.

a Adjusted for: sex, sepsis, EN initiation (h), protein (g/kg/day).

The Week 1 Z-score was considered.

In the adjusted linear regression analysis, WAZ was lower in both groups of male PTNB compared to female PTNB (Supplementary Table II).

## Discussion

This study investigated the behavior of the WAZ in VLBW and ELBW PTNB, comparing them with LBW PTNB admitted to a NICU. A downward trend in WAZ was observed across all phases of hospitalization, which was associated with a significant interaction of two main factors: birth weight and length of hospitalization for both groups. However, in preterm infants with VLBW and ELBW, the reduction in the score was more pronounced and significant during the first week of hospitalization. Despite this, energy and protein intakes increased significantly during this phase of hospitalization for both analyzed groups.

The study also investigated factors associated with the behavior of WAZ. It was observed that male sex, time to initiation of EN, the presence of sepsis, and protein intake explained approximately 20 % of the variability of this score in VLBW and ELBW PTNB compared to LBW PTNB. Male sex was a factor significantly associated with a reduction in WAZ in both groups of PTNB. However, the variability of WAZ attributed to sex was eight times greater in the VLBW and ELBW groups.

As observed in this study, male preterm infants with VLBW and ELBW may be more susceptible to a greater negative deviation in WAZ at the beginning of hospitalization. According to Tottman et al. [14], the body composition of PTNB differs according to sex, with a lower fat mass content observed in males, which results in a lower endogenous supply of energy substrate, exacerbating weight loss and vulnerability, especially in early extrauterine life. Additionally, PTNBs have difficulty absorbing fatty acids due to the immaturity of the gastrointestinal tract [14,20].

In the present study, the presence of sepsis during hospitalization was associated with a significant reduction in WAZ in VLBW and ELBW preterm infants. It is expected that the septic, hypermetabolic state per se would impact this group more intensely, affecting their growth [21]. However, Raban et al. [22], when evaluating the rate of initiation and advancement of nutritional therapy with donor human milk, found no correlation between the occurrence of sepsis and the weight of ELBW PTNB.

In the absence of absolute contraindications, the introduction of EN with human milk can mitigate initial weight loss and improve growth indices in ELBW PTNB [22]. However, the use of formula does not have the same effect, due to the lower bioavailability of nutrients and the absence of immunological and trophic factors found in human milk [21,22]. In the present study, PTNB predominantly received preterm infant formula, and may or may not have received expressed breast milk from their own mothers. Even so, it was observed that EN before 24 h of hospitalization was associated with an increase in WAZ in the VLBW and ELBW group.

A meta-analysis conducted by Kumar et al [23]. investigated the effect of human milk fortified with preterm infant formula compared to unfortified human milk on the growth of VLBW PTNB. Five randomized clinical trials were included, and the results indicated a positive effect of human milk fortification with preterm infant formula, which was superior to that observed with unfortified human milk. Although the authors concluded that the evidence is of low quality, they suggest that fortification of human milk with preterm infant formula may be a safe alternative to promote short-term growth of LBW PTNB in developing countries.

Nevertheless, the authors cannot assert that the observed benefits in the present study were due to human milk, formula, fortifier, or their combination, given that nutritional intakes could be directly linked to the use of formula and fortifiers. This highlights the necessity for future studies that compare preterm infants fed exclusively with human milk to those on infant formula, with the goal of more precisely evaluating the effects of each nutritional intervention on growth outcomes in this context.

In the present study, the VLBW and ELBW PTNB received a high protein intake in the first few days of postnatal life. This result corroborates the indication of the study by Lygerou et al. [24], which evaluated the impact of macronutrient supply on the growth velocity of PTNB, concluding that, as with energy, providing a positive protein balance is essential to achieve better postnatal growth rates and prevent extrauterine growth restriction.

Proteins represent the second largest component of the body and are intensely metabolized in preterm infants due to the high demand for essential and conditionally essential amino acids for protein synthesis [8,24,25]. During the neonatal period, protein deficit can accumulate rapidly in preterm infants, with a reduction of up to 1.5 %/day of body proteins, in contrast to the normally growing fetus, which has a positive protein balance of about 2 %/day [25]. Amino acid intake at a quantity of 3 g/kg/day before 5 days of life can minimize proteolysis, reduce protein deficit, and improve growth, especially in VLBW and ELBW PTNB [25, 26, 27].

The effects of early and high protein supply to PTNB are widely investigated in meta-analysis studies [28]. In the short term, this supply results in greater weight gain, and in the long term, in better neuromotor development at two years of age [27, 28, 29]. Furthermore, PTNB with a lower percentage of fat mass who received a protein intake higher than conventional showed a higher rate of weight recovery and greater accumulation of body fat [28, 29, 30].

In the present study, weight gain velocity was lower in VLBW and ELBW PTNB compared to LBW PTNB; however, it was adequate according to current recommendations [26,27]. The reference for adequate growth of preterm infants is the intrauterine rate, generally 15 to 20 g/kg/day; however, the effort to maintain this rate in the extrauterine environment is an arbitrary practice, particularly for hospitalized PTNB [10,15].

Accelerating the rate of weight gain in PTNB in the neonatal phase can trigger changes in body composition, with a consequent increase in fat mass and altered metabolism [11,15]. In the long term, attention to excessive accumulation of body fat is necessary due to the increased risk of developing chronic diseases such as obesity, hypertension, and diabetes [6,11].

For an adequate evaluation of this rate, it is essential to define the period for obtaining the initial weight, the calculation method, and the time between measurements [19]. In this study, the average between two points, starting from the weight nadir, was used to calculate weight gain velocity. For most preterm infants, the nadir occurred before the first week, with an average of five days of postnatal life. The identification of the weight nadir is valuable information in the context of care and allows excluding the postnatal weight loss phase for the calculation of this rate.

The present results highlight that, in the first week of hospitalization, there was a significant reduction in WAZ at the same time that a significant increase in weight gain velocity was observed. This phenomenon has been described in several studies and corroborates the interpretation that the curves used for calculating the Z-score value were developed for preterm infants in good health conditions [11, 12, 13,15].

Thus, they should be applied to PTNB hospitalized in the NICU as an additional parameter that informs about the distance from the reference median. However, other clinical factors indicative of well-being should be considered, such as clinical stability, weight gain velocity, achievement of energy and nutrient goals, and diet tolerance [11, 12, 13].

This study presents strengths and some methodological limitations. Among the strengths, the sample size of over 250 patients and the use of statistical analyses with intra- and inter-subject adjustments stand out, as both aspects reduce the probability of type I statistical error. As limitations, the high rate of loss to follow-up due to the absence of information in the service records, as well as the absence of sociodemographic and clinical data of the mother, and the limited external validation of the results to population groups similar to those of the present study, should be considered. While the grouping of VLBW and ELBW infants was necessary for analytical feasibility, this approach may have masked specific differences or heterogeneities in outcomes that could exist between the groups if analyzed separately. Therefore, the generalization of these findings to the broader VLBW population should consider this aggregation. Additionally, the absence of data on mechanical ventilation, oxygen therapy, and vasoactive drugs in the nutritional records represents a potential source of confounding, as these factors can directly impact the growth and nutritional status of preterm infants. The authors suggest that future studies with access to more detailed clinical data explore these aspects.

In conclusion, the downward trend of the WAZ curve was associated with the interaction between BW and length of hospitalization in PTNB with VLBW and ELBW. Furthermore, factors such as male sex and the presence of sepsis contributed to the observed decline. Conversely, the initiation of enteral nutrition (EN) before 24 h of hospitalization and a protein intake above 3.5 g/kg/day in the first days of life mitigated the decrease in WAZ. However, these factors were not sufficient to prevent nutritional deficit in VLBW and ELBW PTNB compared to LBW PTNB.

## Conflicts of interest

The authors declare no conflicts of interest.

## Appendix Supplementary materials

Image, application 1

## Footnotes

Supplementary material associated with this article can be found in the online version at doi:10.1016/j.jped.2025.101443.
