# Supplementary material for: Factors associated with weight Z-score in very low birth weight and extremely low birth weight preterm infants during hospitalization
Source: J Pediatr (Rio J). 2025 Sep 25;101(6):101443. doi: 10.1016/j.jped.2025.101443 (PMC12509101; doi:10.1016/j.jped.2025.101443)
Supplement: Supplementary file 1 [file mmc1.docx]

**JPED-D-25-00152_ Supplementary Material**

**Supplementary Table I** Z-scores of growth indices: weight, head circumference, and length-for-age, according to birth weight category and week of hospitalization, of preterm newborns admitted to a Neonatal Intensive Care Unit. University Hospital-UFPEL, 2017-2019.

|  | All (n = 261) | | | LBW (n = 170) | | | VLBW e ELBW (n = 91) | | |  |  |
| --- | --- | --- | --- | --- | --- | --- | --- | --- | --- | --- | --- |
|  |  |  |  |  |  |  |  |  |  |  |  |
|  | Mean | IC 95% | | Mean | IC 95% | | Mean | IC 95% | | *P^*^* |  |
| **Admission** |  |  |  |  |  |  |  |  |  |  |  |
| **Weight** | 0.25 | 0.10 | 0.40 | 0.56 | 0.41 | 0.71 | -0.32 | -0.61 | -0.02 | <0.001 |  |
| **Head circumference** | -0.02 | -0.19 | 0.13 | 0.32 | 0.13 | 0.51 | -0.69 | -0.96 | -0.43 | <0.001 |  |
| **Length** | -0.28 | -0.43 | -0.12 | -0.01 | -0.18 | 0.15 | -0.78 | -1.08 | -0.49 | <0.001 |  |
| Week 1 |  |  |  |  |  |  |  |  |  |  |  |
| **Weight** | -0.60 | -0.75 | -0.44 | -0.34 | -0.49 | -0.18 | -1.05 | -1.34 | -0.75 | <0.001 |  |
| **Head circumference** | -2.12 | -4.19 | -0.06 | -2.35 | -5.98 | 1.27 | -1.83 | -2.14 | -1.51 | <0.001 |  |
| **Length** | -0.18 | -0.38 | 0.02 | 0.14 | -0.09 | 0.38 | -0.62 | -0.95 | -0.30 | <0.001 |  |
| Week 2 |  |  |  |  |  |  |  |  |  |  |  |
| **Weight** | -0.89 | -1.08 | -0.69 | -0.69 | -0.93 | -0.45 | -1.05 | -1.34 | -0.76 | 0.103 |  |
| **Head circumference** | -1.46 | -1.73 | -1.19 | -0.83 | -1.30 | -0.36 | -1.82 | -2.13 | -1.52 | <0.001 |  |
| **Length** | -0.70 | -0.97 | -0.43 | -0.35 | -0.76 | -0.04 | -0.92 | 1.28 | -0.57 | 0.069 |  |
| Week 3 |  |  |  |  |  |  |  |  |  |  |  |
| **Weight** | -1.01 | -1.23 | -0.78 | -0.95 | -1.28 | -0.62 | -1.03 | -1.31 | -0.74 | 0.795 |  |
| **Head circumference** | -1.62 | -1.94 | -1.29 | -1.46 | -2.72 | -0.19 | -1.65 | -1.97 | -1.34 | 0.679 |  |
| **Length** | -0.72 | -1.02 | -0.41 | -0.57 | -1.23 | 0.07 | -0.75 | -1.10 | -0.39 | 0.842 |  |
| Week 4 |  |  |  |  |  |  |  |  |  |  |  |
| **Weight** | -1.08 | -1.40 | -0.77 | -1.36 | -2.09 | -0.63 | -1.04 | -1.39 | -0.69 | 0.511 |  |
| **Head circumference** | -1.89 | -2.34 | -1.45 | -1.70 | -4.87 | 1.47 | -1.92 | -2.34 | -1.51 | 0.509 |  |
| **Length** | -1.04 | -1.45 | -0.63 | -0.90 | -1.86 | 0.05 | -1.06 | -1.52 | -0.60 | 0.824 |  |

* Mann-Whitney U test between weight categories. LBW, Low birth weight; VLBW/ELBW, Very Low/Extremely Low Birth Weight

**Supplementary Table II** Linear regression of Weight Z-score. according to birth weight category and sex. of preterm newborns admitted to a Neonatal Intensive Care Unit. University Hospital-UFPEL. 2017-2019.

|  | **Weight Z-score-** | | | | | | | | | | |  |
| --- | --- | --- | --- | --- | --- | --- | --- | --- | --- | --- | --- | --- |
|  | **Unadjusted analysis** | | | | |  | **Adjusted analysis^a^** | | | | |  |
|  | B | β | IC 95% | R^2^ | *p* |  | B | β | IC 95% | R^2^ | *p* |  |
| **Very Low and Extremely Low Birth Weight** |  |  |  |  |  |  |  |  |  |  |  |  |
| Sex |  |  |  |  |  |  |  |  |  |  |  |  |
| Female | Ref. |  |  |  |  |  | Ref |  |  |  |  |  |
| Male | -0.34 | 0.37 | -0.70; -0.02 | 2.5 | 0.036 | -0.29 | | 0.30 | 0.04;0.54 | 67 | 0.021 |  |
| **Low Birth Weight** | |  |  |  |  |  |  |  |  |  |  |  |
| Sex | |  |  |  |  |  |  |  |  |  |  |  |
| Female | | Ref. |  |  |  |  |  | Ref |  |  |  |  |
| Male | | -0.15 | -0.12 | -0.57;0.33 | 1 | 0.607 | -0.33 | | -0.25 | -0.45;-0.05 | 85 | 0.015 |

B, intercept; β, standardized coefficient; 95% CI, 95% confidence interval; R², adjusted R² (%). N = 177.

^a^ Analysis adjusted for: protein (g/kg/day); Length Z-score; and Head Circumference Z-score.

The Week 1 Z-score was considered.


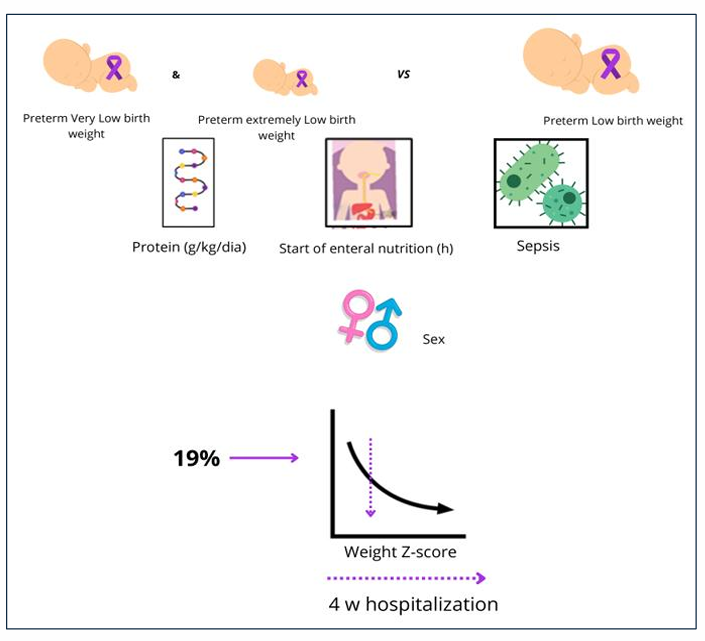


**Supplementary Figure 1** Factors associated with weight Z-score according to sex in preterm newborns admitted to the Neonatal Intensive Care Unit of the University Hospital - UFPEL (N = 261), 2017–2020.
